# Protective Effects of a Probiotic *Lacticaseibacillus paracasei* MSMC39-1 on Kidney Damage in Aged Mice: Functional Foods Potential

**DOI:** 10.3390/foods14111874

**Published:** 2025-05-25

**Authors:** Pol Sookpotarom, Jirapat Namkaew, Kaikwa Wuttisa, Chantanapa Chantarangkul, Praewpannarai Jamjuree, Thiranut Jaroonwitchawan, Malai Taweechotipatr

**Affiliations:** 1 Doctor of Medicine Program, Faculty of Medicine, Srinakharinwirot University, Bangkok 10110, Thailand; pol.sookpotarom@g.swu.ac.th; 2 Futuristic Science Research Center, School of Science, Walailak University, Thasala, Nakhon Si Thammarat 80160, Thailand; namkaew.j@gmail.com; 3 Research Excellence Center for Innovation and Health Products (RECIHP), School of Allied Health Sciences, Walailak University, Nakhon Si Thammarat 80160, Thailand; 4 Center of Excellence in Probiotics, Srinakharinwirot University, Bangkok 10110, Thailand; 5 Department of Microbiology, Faculty of Medicine, Srinakharinwirot University, Bangkok 10110, Thailand; 6 Clinical Research Center, Faculty of Medicine, Srinakharinwirot University, Ongkharak, Nakhon Nayok 26120, Thailand

**Keywords:** aging, chronic inflammation, anti-inflammation, antioxidant, probiotics

## Abstract

Kidney fibrosis and inflammation are significant contributors to the decline in renal function associated with aging. These processes are characterized by structural changes, such as glomerular sclerosis and interstitial fibrosis, which exacerbate kidney injury and inflammation in aged individuals. Probiotics have gained increasing attention for their potential health-promoting effects. However, further investigation is required to fully understand the mechanisms underlying these benefits. We hypothesize that probiotics could ameliorate fibrosis through the immunomodulatory effects of probiotics and by improving kidney tissue inflammation. Sixteen-month-old aging mice were administered *Lacticaseibacillus paracasei* MSMC39-1 for four months compared to young mice (six-month-old) and aged mice (twenty-month-old). The research found that following the administration of probiotic MSMC39-1, there were significant improvements in kidney inflammation, as evidenced by reductions in pro-inflammatory cytokines, fibrosis, and inflammatory cells within the tissue. Moreover, the findings demonstrated that probiotic MSMC39-1 significantly normalized levels of malondialdehyde (MDA), and rescued antioxidant superoxide dismutase (SOD) and glutathione peroxidase (Gpx) in kidney tissue which was consistent with a low mitochondria biogenesis. Further investigations revealed that conditioned medium from MSMC39-1 rescued epithelial kidney cells with damage induced by high glucose. This research provides information and insights into the mechanisms underlying the beneficial health effects of probiotics, offering a deeper understanding of how these probiotics contribute to anti-aging processes in the kidney.

## 1. Introduction

Aging is a natural and complex process that involves a series of biological changes over time. It involves a variety of molecular and cellular changes, such as DNA damage, telomere shortening, and the accumulation of cellular waste products [1]. These are associated with oxidative stress due to the cellular hoarding of reactive oxygen species (ROS), leading to inflammation [2]. Inflammation is characterized by an increase in pro-inflammatory cytokines interleukin 1 β (IL-1β), interleukin 6 (IL-6), and tumor necrosis factor-α (TNF-α), even in the absence of overt infection or injury [3]. This sterile inflammation is believed to play a role in the development of various age-related diseases and affect multiple systems, including the kidneys. Senescent cells in the renal tubular epithelium contribute to renal aging and are associated with increased levels of inflammatory cytokines and fibrosis markers [4,5,6]. The accumulation of senescent cells leads to a pro-inflammatory environment, often referred to as “inflammaging”, which exacerbates kidney damage and fibrosis in aged individuals [5,7]. For example, aberrantly activated lysophosphatidic acid receptor 1 (LPAR1) is associated with chronic inflammation and renal fibrosis in aged kidneys, and blocking it may reduce excessive inflammation in aged mice, suggesting sterile inflammation leading to kidney fibrosis during the aging process [4].

Inflammatory response is a key driving factor in the aging process in the kidneys. Aged mice experienced more severe acute kidney injury (AKI) compared to young mice, with increased pro-inflammatory pathways and cellular senescence [5]. AKI is associated with increased inflammation and cellular senescence, leading to high mortality and morbidity in older patients [7]. The kidney contains both resident and recruited macrophages. Increased macrophage infiltration in aged mice contributes to chronic low-grade inflammation and fibrosis in aged kidneys, with ferroptosis signaling playing a crucial role [6]. Research evidence shows that intestinal microbiota rejuvenation reduces renal interstitial fibrosis and delays renal senescence in aged male mice by inhibiting the NF-κB signaling pathway [8]. Additionally, probiotic supplementation can improve renal function and fibrosis in elderly mice by reducing intestinal inflammation and promoting inflammatory macrophages [9]. Therefore, there is a crosstalk of the gut-kidney axis in maintaining kidney homeostasis and gut microbiota. The gut microbiota undergoes significant changes with age, impacting its diversity and functional capabilities. These shifts may lead to greater gut permeability and an increase in systemic inflammation [10,11].

Probiotics are live microorganisms that, when taken in sufficient quantities, provide health benefits to the host [12]. The most commonly used probiotics include bacteria belonging to the *Lactobacillus* and *Bifidobacterium* genera [13]. *Lactobacillus*, notably, has proven to be an important genus with numerous beneficial biological characteristics. Potentially, *L. reuteri* LR12 and *L. lactis* LL10 illustrate positive scavenging activity of the 2,2-diphenyl-1-picryl-hydrazyl-hydrate (DPPH) free radical, hydroxyl radical, and superoxide radical [14]. More specifically, a mixture of *L. acidophilus* La-14, *L. casei* Lc-11, *L. lactis* Ll-23, *B. lactis* Bl-04, and *B. bifidum* Bb-06 has been shown to lower the concentrations of TNF-α and IL-6 [15]. In this context, *L. paracasei* HII01 has demonstrated the most significant impact on promoting longevity and anti-aging effects in *Caenorhabditis elegans* [16]. Based on our evidence, *Lacticaseibacillus paracasei* (*L. paracasei*) MSMC39-1 (formerly named: *Lactobacillus paracasei* MSMC39-1), previously isolated from neonatal feces, manifested an anti-inflammatory effect on alcohol-induced hepatitis in rats and dextran sulfate sodium-induced colitis in rats [17,18]. Gaining insight into the processes of sterile inflammation and fibrosis in aging kidneys is essential for creating effective treatment strategies. This study aimed to assess the anti-inflammatory and antioxidant effects of the probiotic *L. paracasei* MSMC39-1 in an aging mouse model. Our findings suggest that *L. paracasei* MSMC39-1 may serve as a beneficial anti-aging probiotic, potentially slowing down the aging processes in the kidneys and offering a foundation for supplements aimed at preventing age-related diseases and enhancing gut health.

## 2. Results

### 2.1. L. paracasei MSMC39-1-Derived Conditioned Medium Rescues Kidney Epithelial Cells from Damage

**Figure 1:**
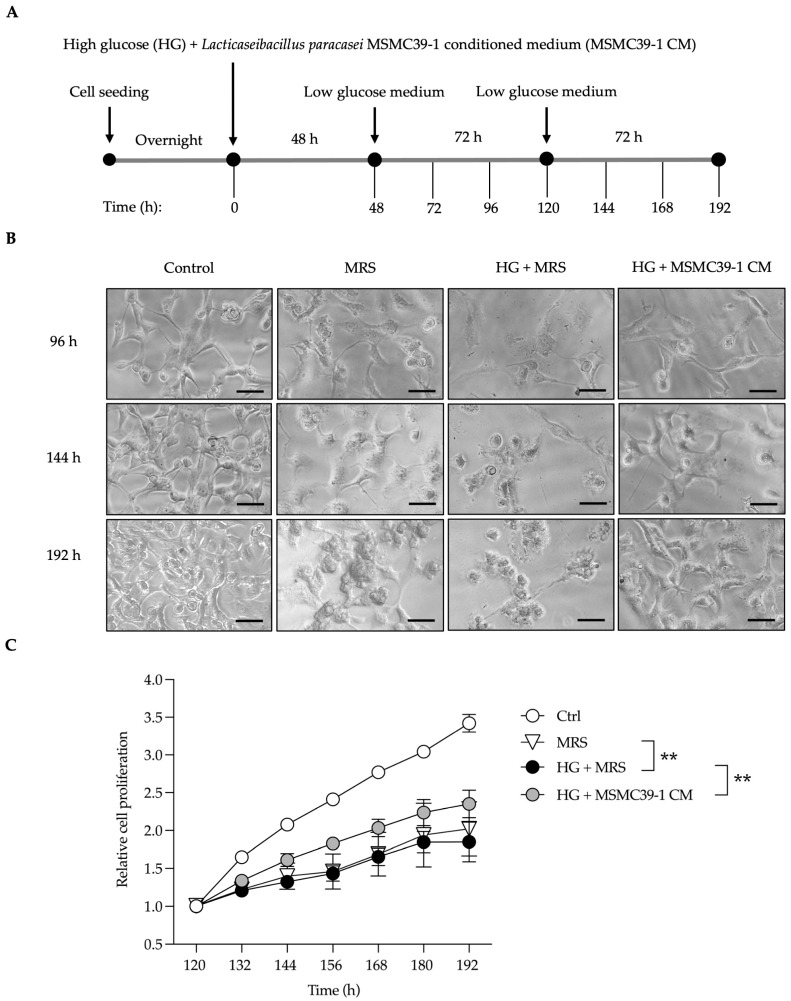
A conditioned medium generated from *L. paracasei* MSMC39-1 protects damaged kidney epithelial cells. (**A**) Schematic representation of the treatment strategy of *L. paracasei* MSMC39-1-derived conditioned medium in kidney epithelial cells. (**B**) Representative microscopic images and (**C**) relative cell proliferation of Vero cells determined using a WST-8 assay. The cells were co-treated with high glucose at 25.5 mM and 1/30 volume of *L. paracasei* MSMC39-1-derived conditioned medium (HG + MSMC39-1 CM) for 48 h followed by low glucose (5.5 mM) medium for another 144 h. De Man, Rogosa, and Sharpe (MRS) broth was used as a vehicle control (HG + MRS). Data are means ± SD (*n* = 4). Statistical analysis was a two-way ANOVA, ** *p* < 0.01 compared to HG + MRS-treated cells. Data are representative of three reproducible and independent experiments. The scale bar is 50 µm.

Aging naturally affects various organs, especially the kidneys, leading to structural and functional decline. This decline is often exacerbated by inflammation and oxidative stress. Additionally, high glucose levels significantly contribute to kidney damage and fibrosis by inducing oxidative stress, inflammation, and activating fibrogenic pathways. We treated an epithelial kidney cell line with high glucose (HG) at 25.5 mM for 48 h with and without conditioned medium (CM) compared to De Man, Rogosa, and Sharpe (MRS) broth control. A fresh medium was added, and the cells were maintained in a low-glucose environment for a duration of 192 h, as illustrated in the experimental schematic (Figure 1A). We examined the morphology of the cells and noted that HG conditions led to a change in cell shape at 96 h, resulting in an irregular appearance (Figure 1B). At 192 h, significant cell shrinkage was evident in the HG treatment. In contrast, the MRS treatment showed only minor alterations in cell morphology at 96 h, but these changes became more pronounced over time (Figure 1B). Importantly, after the introduction of CM for 48 h alongside HG treatment, CM successfully preserved the normal cellular architecture from 96 to 192 h, exhibiting a morphology comparable to that of the control group (Figure 1B). The findings revealed that high glucose treatment led to a decline in cell proliferation, indicating possible cellular damage (Figure 1C). Conversely, epithelial kidney cells exposed to CM demonstrated a significant improvement in the cell proliferation ratio (about 0.7 times) (Figure 1C). Consistently, high glucose conditions lead to cellular damage and reduced proliferation, while CM may help to alleviate some of this damage, though its impact on overall cell proliferation is minimal.

### 2.2. Attenuation of Kidney Damage in Aged Mice by L. paracasei MSMC39-1

**Figure 2:**
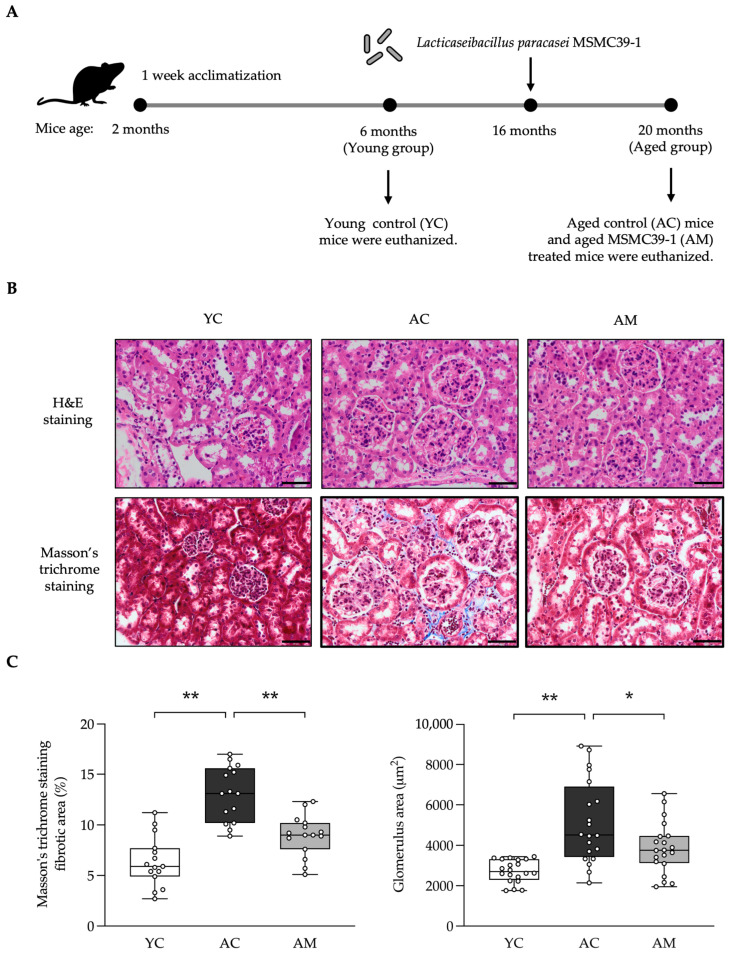
Effects of *L. paracasei* MSMC39-1 on kidney injury in aged mice. (**A**) Schematic representation of the *in vivo* treatment strategy of *L. paracasei* MSMC39-1 in aged mice. (**B**) Representative microscopic images of H&E and Masson’s trichrome staining of mice administered with *L. paracasei* MSMC39-1. (**C**) Quantification of Masson’s trichrome staining (*n* = 15 fibrous areas, *n* = 20 glomeruli) of aged mice administered with *L. paracasei* MSMC39-1 for 4 months (AM). Young mice (6 months of age) were included as a control group (YC). Data are means ± SD (*n* = 5 mice, each containing 3 slices/mouse for Masson’s trichrome staining fibrotic area quantification and 4 glomeruli/mouse for glomerulus area quantification). Statistical analysis was a one-way ANOVA followed by Dunnett’s multiple comparisons test, * *p* < 0.05, ** *p* < 0.01 compared to the aged control (AC) group. The scale bar is 50 µm.

To further determine whether MSMC39-1 could protect kidney damage in aged mice, mice were divided into 3 groups, including normal young control (YC) at 6 months old, aged control (AC) at 20 months old; AC mice were given MSM39-1 beginning at 16months old via oral gavage, called the AM group (Figure 2A). Our analysis of liver tissue through histological examination revealed no signs of liver damage in the AM group after a four-month intervention (Supplementary Figure S1). We assessed kidney pathology using hematoxylin and eosin (H&E) staining, which highlighted the pathological changes in both aged mice and those treated with MSMC39-1. As illustrated in Figure 2B, both the AM group and the YC group displayed normal glomerular architecture, size, and shape, with no signs of hypertrophy, fibrosis, or sclerosis in the kidney tissue. In contrast, the AC group exhibited mild glomerulosclerosis, slight mesangial cell proliferation, and mild glomerular hyperplasia, as shown in Figure 2B. In Figure 2C, there was a marked increase in the glomerular fibrotic area, as evidenced by collagen deposition observed through Masson’s trichrome staining in the AC group relative to the YC group. Furthermore, an increase in glomerular size was noted in the AC group. The administration of MSMC39-1 was found to reverse these changes. Overall, the results indicate that MSMC39-1 supplementation during middle age could mitigate kidney damage related to aging.

### 2.3. Reducing Blood Glucose Levels and Enhancement of Kidney Antioxidant Defense by L. paracasei MSMC39-1

**Figure 3:**
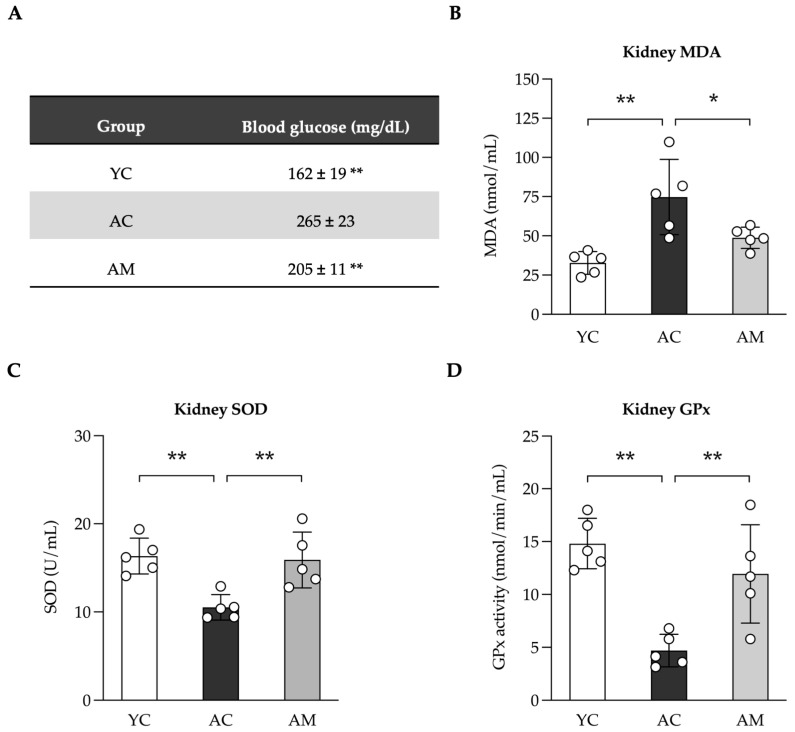
Effects of *L. paracasei* MSMC39-1 on high glucose and oxidative stress in aged mice. (**A**) Serum blood glucose, (**B**) kidney malondialdehyde (MDA) levels, (**C**) kidney glutathione peroxidase (Gpx) levels and (**D**) kidney superoxide dismutase (SOD) levels in aged mice administered with *L. paracasei* MSMC39-1 for 4 months (AM). Young mice (6 months of age) were included as a control group (YC). Data are means ± SD (*n* = 5 mice). Statistical analysis was a one-way ANOVA followed by Dunnett’s multiple comparisons test, * *p* < 0.05 and ** *p* < 0.01 compared to the aged control (AC) group.

Elevated glucose levels, particularly in the context of diabetes or chronic hyperglycemia, play a crucial role in the development of kidney fibrosis, especially among aged individuals. We subsequently assessed blood glucose levels in YC, AC, and AM mice. Our findings indicated that blood glucose was significantly higher in the AC group compared to the YC group. Importantly, the AM exhibited a notable reduction in blood sugar levels when compared to AC (Figure 3A). Additionally, we evaluated kidney lipid peroxidation by measuring MDA, an indicator of oxidative stress. The results showed that MDA levels were highest in the AC group, returning to normal following MSMC39-1 treatment in middle-aged mice, while MDA levels were elevated in AC compared to YC (Figure 3B). GPx and SOD are vital in alleviating oxidative stress and protecting cells from DNA damage. Following this, we measured the activities of SOD and GPx in kidney tissue. Our analysis showed that SOD (Figure 3C) and GPx (Figure 3D) activities were significantly lower in the AC group compared to the YC group. However, in the AM group, these activities were restored, indicating that MSMC39-1 possesses robust antioxidant properties. The findings from the MSMC39-1 treatment suggest that it can effectively improve both blood glucose levels and oxidative stress when compared to the aging group, aligning more closely with the young group.

### 2.4. Improving of Kidney Mitochondria DNA (mtDNA) Content in Aged Mice by L. paracasei MSMC39-1

**Figure 4:**
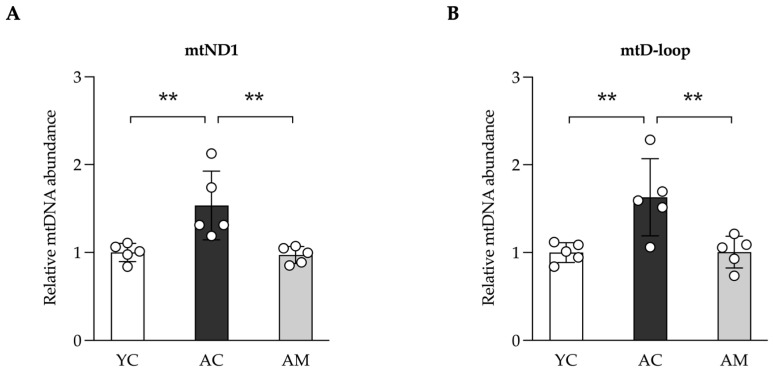
Improvements of kidney mitochondria DNA (mtDNA) content in aged mice by *L. paracasei* MSMC39-1. (**A**) qPCR analysis of kidney mtND1 and (**B**) mtD-loop in aged mice administered with *L. paracasei* MSMC 39-1 for 4 months (AM). Young mice (6 months of age) were included as a control group (YC). Data are means ± SD (*n* = 5 mice). Statistical analysis was a one-way ANOVA followed by Dunnett’s multiple comparisons test, ** *p* < 0.01 compared to the aged control (AC) group.

The levels and integrity of mtDNA are frequently affected by the cumulative impacts of aging, oxidative stress, and various age-related factors [19]. In our study, we assessed the mtDNA content in kidney tissue, which encompasses the total mtDNA present. The results revealed that kidneys from the AC group displayed a significantly elevated copy number of both mitochondrial NADH dehydrogenase 1 (mtND1) and mitochondrial displacement loop (mtD-loop) (Figure 4A,B) in contrast to the relatively lower levels observed in the YC group. Following the administration of MSMC39-1, mtDNA levels in the AM group were restored to those typical of the YC group. This suggests that MSMC39-1 may serve as a potential agent for normalizing mtDNA levels in aged kidney tissue.

### 2.5. Anti-Inflammation in Kidney by L. paracasei MSMC39-1

**Figure 5:**
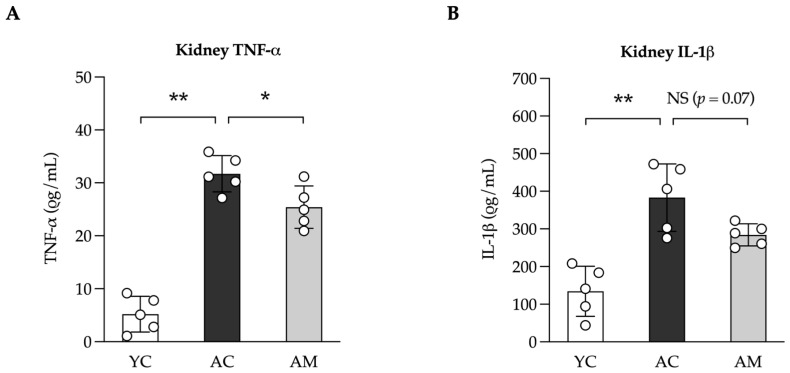
Anti-inflammatory effects in the kidneys of aged mice administered with *L. paracasei* MSMC39-1. (**A**) Kidney levels of TNF-α and (**B**) IL-1β in aged mice administered with *L. paracasei* MSMC39-1 for 4 months (AM). Young mice (6 months of age) were included as a control group (YC). Data are means ± SD (*n* = 5 mice). Statistical analysis was a one-way ANOVA followed by Dunnett’s multiple comparisons test, * *p* < 0.05, ** *p* < 0.01, and NS = not significant compared to the aged control (AC) group.

The administration of MSMC39-1 has demonstrated a notable capacity in this study to reduce pro-oxidative stress and fibrosis in the kidneys. The interplay between oxidative stress and inflammation may contribute to the development of tissue fibrosis. Consequently, we assessed the levels of the inflammatory cytokines TNF-α and IL-1β in kidney tissue. In the AM group, there was a significant reduction in TNF-α and a slight decrease in IL-1β levels compared to the AC group (Figure 5A,B).

### 2.6. Positive Effects of L. paracasei MSMC39-1 on Maintained Colon Integrity and Anti-Oxidative Stress

**Figure 6:**
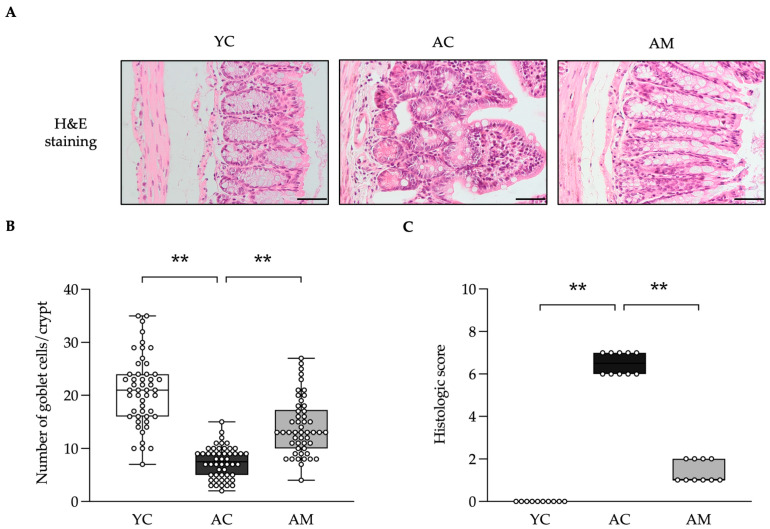
Improvements of colon damage in aged mice administered with *L. paracasei* MSMC39-1. (**A**) Representative microscopic images of H&E staining, (**B**) quantification of colonic goblet cell number in crypts (*n* = 50) and (**C**) histological score of colon damage (*n* = 10) in aged mice treated with *L. paracasei* MSMC39-1 for 4 months (AM). Young mice (6 months of age) were included as a control group (YC). Data are means ± SD (*n* = 5 mice, each containing 10 crypts/mouse for colonic goblet number quantification and 2 slices/mice for the histological score assessment). Statistical analysis was a one-way ANOVA followed by Dunnett’s multiple comparisons test, ** *p* < 0.01 compared to the aged control (AC) group. The scale bar is 100 µm.

The interplay between the kidneys and the gut is significant, as they collaboratively contribute to the maintenance of homeostasis and the prevention of inflammation and oxidative stress at both local and systemic levels [20]. We hypothesized that MSMC39-1 might safeguard against alterations in gut architecture induced by aging, which could lead to inflammation in the kidneys. The length of the colon exhibited a slight variation, with the AC group being shorter than the YC group, while the length in the AM group could be normalized to the YC group (Supplementary Figure S2A). Subsequently, we assessed the gut histology using the histological score presented in Table 1. across three groups: AC, YC, and AM. The AM group demonstrated a reduction in age-associated intestinal changes; inflammatory conditions and the colonic mucosa were preserved, exhibiting uniform colonic tissue and epithelial cells with normal microvilli and crypt structures, along with the lamina propria showing minimal chronic inflammatory cell infiltration (Figure 6A). These findings were comparable to those observed in the YC group (Figure 6A). In contrast, the colonic tissue of the AC group displayed areas with depleted goblet cells, structural loss of microvilli and crypts (Figure 6A). The reduction in the number of goblet cells was clearly observed in the AC group, whereas the AM group appears to reverse this decline (Figure 6B). Furthermore, inflammatory cell infiltration was evident throughout the lamina propria, submucosa, and deeper layers (Figure 6A). Histological grading indicated that the probiotic MSMC39-1 significantly improved the age-associated intestinal changes compared to the AC group, as illustrated in Figure 6C. The assessment of oxidative stress in the colon was conducted through the measurement of MDA levels, revealing an elevation in the MDA levels in the AC group, whereas the MDA levels were normalized in the AM group, indicating that the observed age-associated intestinal changes may be influenced by oxidative stress (Supplementary Figure S2B). In summary, MSMC39-1 seems to be a promising target for preserving gut architecture and exerting anti-oxidative stress effects in aged mice.

## 3. Discussion

Aging is associated with physiological alterations in the body that can result in diminished functionality and the emergence of diseases, such as dementia, cardiovascular ailments, diabetes mellitus, and several chronic conditions, including kidney diseases [21]. Probiotics are one of the safest supplements that have the potential to protect or restore the aging process. Probiotics have been suggested to relieve systemic cytokine markers and improve antioxidant levels, resulting in anti-aging effects [22]. The kidney plays a vital role in the aging process and is susceptible to the effects of oxidative stress and inflammation. This study investigates the kidneys of aging mice, based on a model created by Flurkey et al. (2007) [23]. In this model, young mice are represented by those aged 3–6 months, while the aging group consists of mice aged 18–24 months [23]. This is consistent with the ages of the mice in our study. The probiotic MSMC39-1 was administered to mice starting in middle age and continued throughout their old age. The results indicated that treatment leads to significant improvements in oxidative stress, inflammation, and fibrosis in the kidneys of aging mice, highlighting the important roles of probiotics in anti-oxidant and anti-inflammation.

Kidney fibrosis is a common pathological condition linked to aging, characterized by the excessive buildup of extracellular matrix components, which results in tissue scarring and diminished renal function. Our research demonstrates that MSMC39-1 effectively prevents collagen accumulation associated with aging through the contribution of inflammation and oxidative stress in aged mice. However, our study focused on male mice, allowing for a more controlled analysis by avoiding hormonal fluctuations seen in females, especially as they age and experience hormonal declines that can affect inflammation. Previous research has shown gender differences in kidney aging in animal models, with variations in susceptibility to fibrosis, inflammation, and functional decline. In mice, aging kidneys show sex-specific changes in epigenetic markers and injury responses. Female mice exhibit more significant changes in kidney and body weight, serum creatinine, and epigenetic enzyme activity, while 24-month-old male mice show higher levels of H3 damage markers and more severe histological changes with age [24]. Other studies comparing aged male and female mice at 24 months have revealed a unique senescence marker, p21, and SA-β staining in male tubular epithelial cells, while female mice produce significantly higher levels of the inflammatory cytokine IL-1β in their kidneys [25]. Therefore, further research involving female models is crucial to determine if the mechanisms and therapeutic implications we identified apply to both sexes.

MSMC39-1 inhibits the production of inflammatory cytokines, highlighting the connection between kidney inflammation and kidney damage. In agreement, *L. paracasei* HY7207 has shown efficacy in reducing hepatic steatosis, inflammation, and fibrosis in a mouse model of non-alcoholic fatty liver disease (NAFLD) [26]. While the study focused on liver health, the mechanisms by which *L. paracasei* reduces inflammation and fibrosis in the liver could potentially be applicable to the kidneys and other tissues. This suggests the systematic effect of probiotic supplementation in a mouse model. Chronic kidney disease (CKD) is associated with systemic inflammation and oxidative stress in the kidneys, which may result in various complications and a decline in renal function [27]. As a result, the findings align with the work of Wagner et al. (2022), indicating that dysbiosis facilitates the colonization of bacteria that produce urease, indole, and p-cresol-forming enzymes, ultimately leading to the accumulation of uremic toxins [28]. We did not measure those toxic agents produced in aged kidneys, but tissue inflammation is probably due to the accumulation of toxic agents in the aged organ in our model. Instead, we found that blood glucose is increased in aged mice; this is likely to be due to a damaged molecule in aged mice that leads to a systemic effect. In addition, studies indicate that the use of *L. paracasei* and *L. plantarum* can lead to improved kidney function and a reduction in inflammation, potentially serving as a preventive measure against the progression of chronic kidney disease [29]. This is achieved through the enhancement of the downregulation of harmful metabolites, such as uremic toxins. Likewise, probiotics develop natural defenses that inhibit harmful bacteria, including those that generate uremic toxins. Recent research has demonstrated that the administration of probiotics can modify the intestinal microbiota in aging mice, resulting in improved outcomes for both acute and chronic kidney injuries [9]. MSMC39-1 supplementation exhibits an anti-inflammatory effect by reducing pro-inflammatory cytokine levels of IL-1β and TNF-α in aged kidney mice. Similarly, colon histology score, immune cell infiltration, crypt length distortion, and microvilli damage in aged mice are also rescued by MSMC39-1 supplementation. This effect underscores probiotic properties such as immune system modulation via communication between the renal and gut system, which is known as the gut–kidney axis. Moreover, strains of *L. paracasei* have demonstrated the ability to enhance the integrity of the gut barrier by elevating the levels of tight junction proteins and mucin, thereby contributing to the reduction of inflammation and the preservation of gut health [30,31]. This is crucial in preventing conditions like colitis and other inflammatory bowel diseases [30,31]. *L. paracasei* X11 supplementation has been shown to lower serum uric acid levels and reduce renal inflammation, which is beneficial in preventing chronic kidney disease [32]. This suggests a protective role in the gut-kidney axis.

Aging in the kidneys is characterized by increased inflammation and oxidative stress, which further promotes fibrosis. This can be achieved through several mechanisms, including NF-κB, along with the production of reactive oxygen species (ROS) [33,34]. Our research indicates that the restoration of pro-inflammatory cytokine expression in the kidneys of aged mice may be attributed to the inhibitory influence of NF-κB activation, as both TNF-α and IL-1β are recognized as well-characterized target genes of NF-kB. Nevertheless, other transcription factors and pathways could also contribute to the reduction of TNF-α and IL-1β. These mechanistic validations are critical to definitively establish NF-κB’s role and will prioritize them in follow-up work. Moreover, we were unable to establish a clear link between oxidative stress and inflammation, as both factors may exacerbate each other in a feedback loop. An imbalance between the production and neutralization of ROS results in the onset of oxidative stress [35]. As a result, an overabundance of hydroxy radicals can damage cell membranes, resulting in lipid peroxidation. This process subsequently elevates the levels of MDA in aging kidneys. This study revealed that MSMC39-1 reduces MDA levels while enhancing the activities of SOD and GPx in the kidney. The improvement of tissue injury may be associated with the levels of antioxidants. In alignment with the findings of Oguntoye et al. (2023), it was observed that Wistar rats receiving provitamin A cassava hydrolysate in conjunction with *L. rhamnosus* exhibited a notable enhancement in antioxidant biomarkers within their kidneys, heart, and liver [36]. In individuals with CKD, the use of probiotics, prebiotics, and synbiotics has been associated with improvements in markers of oxidative stress, such as elevated glutathione (GSH) and total antioxidant capacity (TAC); however, the specific impacts on GPx and SOD remain unspecified [37]. This indicates the potential for probiotics to enhance overall antioxidant capacity, which could indirectly benefit kidney function. The ability of probiotic MSMC39-1 to neutralize free radicals at the cellular and tissue levels is apparent. This aligns with the findings of Lin et al. (2022), which showed that the combination of *Lactobacillus* and *Bifidobacterium* probiotics elevates SOD, catalase and GPx levels in the brain and liver, while decreasing MDA levels in mice undergoing natural aging [38].

Findings from a population-based study suggest that higher levels of fasting glucose are related to glomerular hyperfiltration and microalbuminuria in middle-aged adults, in contrast to insulin resistance measures, which do not exhibit such a connection [39]. Our research indicated that aged mice exhibited an increased blood glucose level, which aligns with kidney damage, and MSMC39-1 supplementation normalizes glucose levels and kidney damage. We hypothesize that elevated glucose levels may contribute to the deterioration of aged kidney tissue. However, the precise mechanisms remain unclear. We used a high glucose concentration of 25.5 mM to induce cellular damage and assessed the effect of conditioned medium from probiotics on cellular damage in vitro. According to the existing literature, one study demonstrated that a high glucose concentration of 25 mM induces sustained expression of p21 (a senescent cell marker) after 24 and 48 h in both human embryonic kidney cells (HEK-293) and primary mouse tubular cells [40]. Another prior study indicated that treatment with high glucose at 25 mM for 36 h resulted in reduced proliferation of human proximal tubule epithelial cells (hPTEC), suggesting that high glucose levels induce cellular injury leading to cell death [41].

Our in vitro investigation utilizing Vero cells as a model indicates that the conditioned medium from MSMC39-1 alleviates cellular damage caused by high glucose exposure. Although Vero cells, which are derived from monkeys, may exhibit different responses to injury compared to human renal cells, they possess solely epithelial characteristics. However, they do not entirely replicate the intricate nature of human kidney tissue. The use of Vero cells allowed for a controlled and reproducible environment to assess glucose-induced damage in kidney epithelial cells, which may provide insights for future mechanistic research in more physiologically relevant models. For instance, three-dimensional cultures of HK2 cells and iPSC-derived proximal tubule-like cells more accurately reflect native cellular functions and gene expression [42,43], thereby offering improved platforms for investigating age-related injuries and evaluating potential therapeutic interventions.

How MSMC39-1 has an effect on improving inflammation and oxidative stress in aged kidneys remains elusive. We speculated that probiotic effects could maintain mitochondrial homeostasis in aged kidneys, which leads to a reduction in inflammation and oxidative stress. We observed that in aging kidneys, mtDNA levels are elevated, correlating positively with inflammation, oxidative stress, and histological changes. While this finding suggests a potential link between mitochondrial homeostasis and probiotic-mediated kidney injury protection, the current data do not elucidate the underlying mechanism. Based on the existing literature, we propose that differences in mtDNA copy number could reflect one or more underlying mechanisms. One possibility is that probiotics may enhance the clearance of damaged mitochondria by promoting mitophagy. Prior studies have demonstrated that the probiotics *Saccharomyces boulardii* and *Lactococcus lactis* can trigger mitophagy by directly activating PRKN/parkin-mediated pathways. This activation boosts the recruitment of PRKN to mitochondria, promotes phospho-ubiquitination, and improves the lysosomal degradation of damaged mitochondria by removing unhealthy mitochondria from *Drosophila* cells exposed to mitochondrial stressors [44]. Another possibility is that aging kidneys seem to activate compensatory mechanisms to reduce mitochondrial damage by enhancing the production of new mitochondria, thereby maintaining cellular energy and function, as indicated by an increase in mtDNA levels. Research indicates that a reduction in cytochrome c levels within kidney mitochondria plays a significant role in the impairment of oxidative phosphorylation (OXPHOS) in older rats. This decline is essential for the process of OXPHOS and results in diminished mitochondrial function in the kidneys of aged rats [45]. Moreover, aging is associated with a reduction in mitochondrial mass and function, which exacerbates renal fibrosis and cellular senescence, primarily through the activation of Wnt/catenin/RAS signaling pathways that are implicated in age-related renal fibrosis [46]. Future studies are required to evaluate how the gut microbiome in the aged gut and kidney is being altered by supplementation with MSMC39-1. In addition, the way in which MSMC39-derived metabolites involve mitigating inflammatory and oxidative stress in aged kidneys also needs to be addressed.

## 4. Materials and Methods

### 4.1. Vero Cell Culture

The African green monkey kidney cell line, known as Vero cells (CCL-81), was obtained by Dr. Sakdiphong Punpai, Innovative Learning Center and Center of Excellence in Medical and Environmental Innovative Research (CEMEIR), Srinakharinwirot University, Bangkok, Thailand. Cells were regularly maintained in DMEM/low glucose (Hyclone, Logan, UT, USA) and supplemented with 10% fetal bovine serum and penicillin-streptomycin (Hyclone, USA). The Vero cells were incubated at 37 °C in a humidified incubator continuously flushed with a mixture of 5% CO_2_ and 95% air.

### 4.2. Probiotic Strain, Culture Conditions, and Conditioned Medium Preparation

A probiotic *L. paracasei* MSMC39-1 strain was sourced from the Center of Excellence in Probiotics, Faculty of Medicine, Srinakharinwirot University, Bangkok, Thailand. The probiotics were cultivated in de Man-Rogosa-Sharpe (MRS) medium (HiMedia Lab., Mumbai, India) at 37 °C under anaerobic conditions for 48 h. The bacterial suspension was adjusted to a density of 10^9^ colony-forming units (CFU) using phosphate-buffered saline, and then administered orally to the mice. For the in vitro experiment, the culture supernatant was centrifuged at 4000 rpm for 10 min at 4 °C. Then, the supernatant was filtered through a 0.22 µm filter and stored at −80 °C for later use as a conditioned medium.

### 4.3. In Vitro Cell Damaging Induced by Glucose Experiment

Vero cells were seeded in a 96-well plate at a density of 2,000 cells/100 µL and incubated overnight. The cells were treated with high glucose (Sigma, Livonia, MI, USA) at 25.5 mM with and without 1/30 volume of conditioned medium for 48 h. The medium was then replaced with fresh low-glucose (5.5 mM) medium, and the cells were maintained until day 8, with the medium changed every 3 days. Throughout this period, the cells were monitored and photographed using a Matero TL Digital Transmitted Light Microscope (Leica Microsystems, Wetzlar, Germany). Cell proliferation was assessed using the WST-8 (MedChem Express, South Brunswick Township, NJ, USA) assay at 460 nm absorbance with a Multiskan SkyHigh (Thermo Scientific, Waltham, MA, USA).

### 4.4. Mitochondrial DNA Measurement

Total cellular DNA was extracted from kidney tissue using GF-1 Tissue DNA Extraction (Vivantis, Selangor, Malaysia) according to the manufacturer’s instructions. Ten nanograms of total cellular DNA was used as a template for mitochondria DNA amplification by real-time PCR with the following sequences encoded for mtDNA ND1 (mtND1) 5′ CTAGCAGAAACAAACCGGGC 3′, 5′ CCGGCTGCGTATTCTACGTT 3′; mtDNA D-loop (mtD-loop) 5′ AATCTACCATCCTCCGTGAAACC 3′, 5′ TCAGTTTAGCTACCCCCAAGTTTAA 3′. Total cellular mtDNA was normalized using endogenous genomic HK2; 5′ GCCAGCCTCTCCTGATTTTAGTGT 3′, 5′ GGGAACACAAAAGACCTCTTCTGG 3′.

Total cellular mtDNA was quantified by ExcelTaq™ 2X Fast Q-PCR Master Mix (SYBR, ROX) (SMBIO, New Taipei City, Taiwan) using the StepOnePlus™ Real-Time PCR System (Applied Biosystems, Foster City, CA, USA). The relative abundance of total mtDNA level was calculated using = 2^(−∆∆Cq)^ according to the reference method [47].

### 4.5. Experimental Animals

Two-month-old male C56BL/6NJcl mice (*Mus musculus*) were purchased from Nomura Siam International Co., Ltd. (Bangkok, Thailand). Animal experiments were carried out according to the guidelines of the Ethics and Research Standardization Section, Srinakharinwirot University (approval number: COA/AE-015-2564). In short, all mice were housed in a room under controlled temperature and humidity ranges of 22 ± 2 °C and 55 ± 10%, respectively, on a 12 h light–dark cycle with free access to food and water. After 1 week of acclimatization, mice were randomly divided, according to the design of the aim of this study, into three groups: (1) young group aged 6 months, (2) aging group aged 20 months, and (3) MSMC39-1 group aged 20 months. Each group was assigned a total of five mice, based on previous studies involving aging mice [48]. The MSMC39-1 group was given 100 µL of MSMC39-1 via oral gavage at 10^9^ CFU/mL for 4 months.

### 4.6. Organ Collection and Preparation

The mice were euthanized using isoflurane anesthesia. Briefly, their abdomens were opened, and the diaphragm barrier between the lungs and liver was cut to reach the heart for excision; then the mice died peacefully without pain. After that, the kidney, colon, and liver were collected. The organs were stored at −80 °C. The fifty to one hundred mg of kidney was homogenized in 500 µL of 10% (*w*/*v*) RIPA buffer and sonicated using an ultrasonic homogenizer (Sonoplus HD 2070; Bandelin, Germany) at 25-30% power for 3 min. All procedures were performed on ice. Thereafter, the homogenates were centrifuged at 15,000× *g* for 10 min at 4 °C. The supernatant was collected and stored at −80 °C for inflammatory cytokines measurement and oxidative stress study. Histopathological analysis: the samples were fixed in 4% (*w*/*v*) paraformaldehyde for 48 h. The kidney was sliced to a 4 µm thickness. The colon was sliced to a 5–7 µm thickness. The liver was sliced to a 5–7 µm thickness. All organs were prepared for further study.

### 4.7. Blood Glucose Evaluation

The blood sample was collected by heparinized blood and centrifuged at 1500× *g* for 15 min at 4 °C. Total sugar was measured using plasma by the Professional Laboratory Management Corp, Co., Ltd., Bangkok, Thailand.

### 4.8. Inflammatory Cytokines Evaluation by ELISA

The inflammatory cytokines, TNF-α and IL-1β, were measured using the mouse TNF-α DuoSet ELISA (#DY410-05, Minneapolis, MN, USA) and the Mouse IL-1β/IL-1F2 DuoSet ELISA (#DY401-05, Minneapolis, MN, USA). The procedures were carried out according to the manufacturer’s protocols. Briefly, the ELISA plate (#DY990, R&D Systems) was coated with capture antibody overnight at 4 °C. After washing the plate, reagent diluent was added for blocking and incubated for 2 h at room temperature. Next, the wells were washed, and supernatant (100 µL/well) was added overnight at 4 °C. Each supernatant was washed off and incubated with the detecting antibody for 2 h at room temperature. The streptavidin-HRP and substrate were added to start the reaction. After that, the wells were treated with stop solution and read at 450 nm using a Biochrom microplate reader (Anthos 2010, Salzburg, Austria). The concentrations of TNF-α and IL-1β were calculated using a standard curve obtained from the TNF-α and IL-1β (mouse) ELISA standard following the manufacturer’s protocols.

### 4.9. Lipoperoxidation and Anti-Oxidative Stress Evaluation

The oxidative markers, including MDA, SOD and GPx were measured using TBARS Assay Kit (#10009055), Superoxide Dismutase Kit (#706002), and Glutathione Peroxidase Assay Kit (#703102) from Cayman Chemical Company, Ann Arbor, Michigan according to the manufacturer’s protocols. The specific absorbance was read using a Biochrom microplate reader (Anthos 2010, Salzburg, Austria). Each enzyme activity was measured by following the manufacturer’s specific instructions and using a standard curve for quantification.

### 4.10. Histopathological Analysis

The kidney and colon tissues were labeled with H&E staining. In addition, kidney tissue was labeled with Masson’s Trichrome for fibrosis. The stained sections were observed and examined under a light microscope (Olympus BX53, Olympus Corporation, Tokyo, Japan). The positive areas of Masson’s Trichrome staining, glomerulus area, and number of goblet cells were analyzed using ImageJ software 1.54i 03 March 2024 version.

For histological scoring of the colon, the sections were graded by two independent investigators blinded to the different cohorts. Histological damage was scored using a protocol described previously [49]. The score ranged from 0 to 10 (total score), which represents the sum of the scores from inflammatory cell infiltration (0–3), tissue damage (0–5), and edema (0–2), as explained in Table 1.

### 4.11. Statistical Analysis

The data were analyzed using GraphPad Prism 9 software and are shown as means ± SD. The statistical analysis of the data was carried out using a one-way or two-way analysis of variance (ANOVA). Values of *p* < 0.05 and 0.01 were considered statistically significant.

## 5. Conclusions

Inflammation and oxidative stress are closely associated with the aging process and are key drivers of tissue damage, including kidney damage. The research demonstrates that probiotic MSMC39-1 exerts a preventive effect in an aging mouse model, likely due to its ability to improve gut structural changes and reduce gut oxidative stress in aging mice. This protective effect subsequently reduces pro-inflammatory cytokines and improves the antioxidant system in aged kidneys, leading to improved kidney damage. Our findings suggest that MSMC39-1 may play a crucial role in establishing new gut homeostasis in aging mice, targeting kidney damage.

## Figures and Tables

**Table 1:** Histological assessment of colon damage.

Feature Graded	Grade	Description
Inflammatory cell infiltration (0–3)	0	Less than three inflammatory cells per field of view at 40× magnification in the lamina propria
	1	Greater than three inflammatory cells per field of view in the lamina propria
	2	Confluence of inflammatory cells extending into the submucosa
	3	Confluence of inflammatory cells present in all tissue layers
Tissue damage (0–5)	0	Normal
	1	Damage limited to the epithelium
	2	Focal ulceration limited to the mucosa
	3	Focal transmural inflammation and ulceration
	4	Extensive transmural ulceration and inflammation bordered by normal mucosa
	5	Extensive transmural inflammation and ulceration involving the entire section
Edema (0–2)	0	No edema
	1	Focal submucosal edema
	2	Extensive submucosal edema involving the entire section

## Data Availability

The original contributions presented in the study are included in the article/Supplementary Material, further inquiries can be directed to the corresponding authors.

## Supplementary Materials

The following supporting information can be downloaded at: https://www.mdpi.com/article/10.3390/foods14111874/s1, Figure S1: Representative microscopic images of H&E staining of the liver sections in aged mice administered with *L. paracasei* MSMC39-1 for 4 months (AM); Figure S2: Effects of *L. paracasei* MSMC39-1 on colon length and oxidative stress in aging mice.
