# Supplementary material for: Protective Effects of a Probiotic Lacticaseibacillus paracasei MSMC39-1 on Kidney Damage in Aged Mice: Functional Foods Potential
Source: Foods. 2025 May 25;14(11):1874. doi: 10.3390/foods14111874 (PMC12154323; doi:10.3390/foods14111874)
Supplement: Supplementary file 1 [file foods-14-01874-s001.zip › foods-3625730-supplementary.pdf]

Supplementary Materials

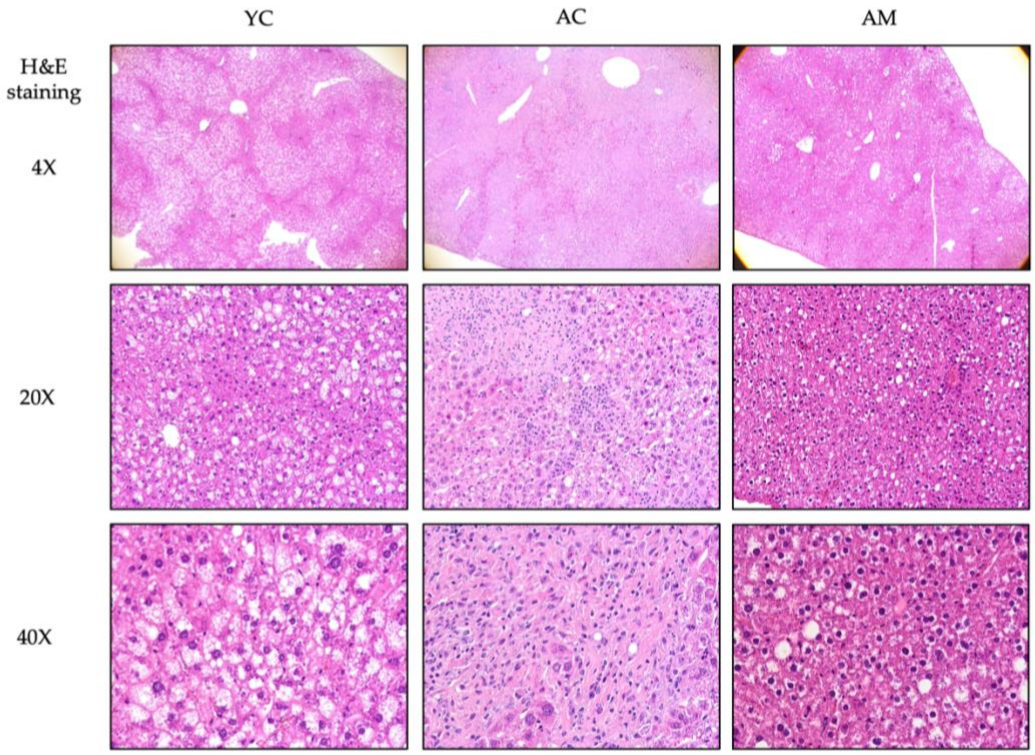

Figure S1. Representative microscopic images of H&E staining of the liver sections in aged mice administered with *L. paracasei* MSMC39-1 for 4 months (AM). Young mice (6 months of age; YC) and aged mice (20 months of age: AC) were included as a control group. Images are representative of liver sections from three mice.

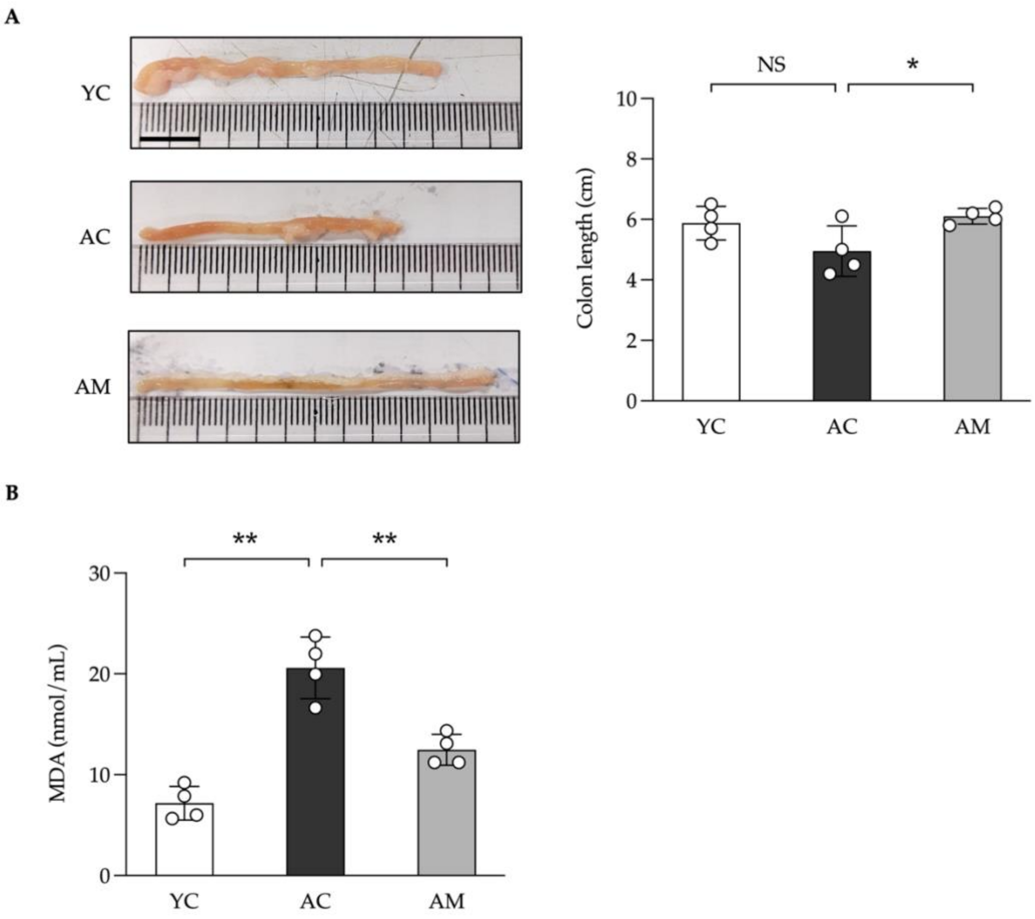

Figure S2. Effects of *L. paracasei* MSMC39-1 on colon length and oxidative stress in aging mice. **(A)** Representative images and quantification of the colon length and **(B)** colon malondialdehyde (MDA) levels in aged mice administered with *L. paracasei* MSMC39-1 for 4 months (AM). Young mice (6 months of age) were included as a control group (YC). Data are means ± SD (n = 4 mice). Statistical analysis was a one-way ANOVA followed by Dunnett's multiple comparisons test, *$p < 0.05$ and **$p < 0.01$ compared to the aged control (AC) group. The scale bar is 1 cm.
